# Dietary squid paste supplementation promotes feed intake via brain-gut dynamic response in Chinese soft-shelled turtle *Pelodiscus sinensis*

**DOI:** 10.7717/peerj.9031

**Published:** 2020-04-24

**Authors:** Cunxin Sun, Yu Qian, Wenbin Liu, Weina Xu, Kaizhou Wang, Bo Liu

**Affiliations:** 1 Key Laboratory of Freshwater Fisheries and Germplasm Resources Utilization, Ministry of Agriculture and Rural Affairs, Freshwater Fisheries Research Center, Chinese Academy of Fishery Sciences, Wuxi, China; 2 Laboratory of Aquatic Nutrition and Ecology, College of Animal Science and Technology, Nanjing Agricultural University, Nanjing, China; 3 School of Agriculture and Biology, Shanghai Jiaotong University, Shanghai, China

**Keywords:** Squid paste, *Pelodiscus sinensis*, Feed intake, Rice protein concentrate, Gut-brain peptide

## Abstract

**Background:**

As the primary source of protein for aquaculture, fishmeal has reached the extremity of sustainable development, our previous studies have proven that rice protein concentrate and squid paste are outstanding protein source and stimulant for *Pelodiscus sinensis*. However, little attention has been given to the molecular mechanism of the appetite modulated by the dietary nutrient factor, especially for a reptile. Thus, the present study aimed to evaluate feed intake and brain-gut dynamic responses to dietary rice protein concentrate and squid paste in Chinese soft-shelled turtle *Pelodiscus sinensis*.

**Methods:**

Three isonitrogenous and isoenergetic practical diets were formulated including 60% fishmeal (CT), 42% fishmeal + 18% rice protein concentrate (RP) and 42% fishmeal + 18% rice protein concentrate + 1% squid paste (RPS), respectively. Microcapsule lysine was supplemented in RP and RPS diets to balance the amino acid profile. Turtles (initial weight 30.65 ± 0.97 g) were fed three times daily to apparent satiation. After the 8-week feeding trial, the turtles were exposed to 48h food deprivation, then the dynamic expression of the orexigenic and anorexigenic peptides were measured.

**Results:**

The results showed that no significant effect was observed on feed intake when fishmeal was replaced by rice protein concentrate (*P* = 0.421), while significantly improved feed intake was found by squid paste supplemented (*P* = 0.02). The mRNA expression of anorexigenic peptides, such as leptin receptor, insulin receptor, pro-opiomelanocortin, cocaine and amphetamine-regulated transcript, cholecystokinin (and its receptor) and glucagon-like peptide-1 receptor in the brain increased significantly at 3 h past feeding (*P* < 0.05), and then decreased. Nevertheless, neuropeptide Y and peptide YY mRNA expression showed the valley at 3h and peak at 12h past feeding. Intestinal cholecystokinin receptor and glucagon-like peptide-1 receptor mRNA expression showed no difference during the postprandial time (*P* > 0.05). The results suggested that squid paste is an outstanding stimulant for *Pelodiscus sinensis*. Furthermore, the orexigenic and anorexigenic peptides evaluated here might play an essential role in short-term fasting to this species, of which the dynamic expression levels were regulated by squid paste.

## Introduction

According to FAO (2014), 23,000,000 excess tons of aquatic production was needed to meet current consumption by 2030. Nevertheless, as the primary source of protein for aquaculture, fishmeal has reached the limit of sustainable development (Tacon & Metian, 2009a; Tacon & Metian, 2009b). Hence, considerable efforts have been made to overcome this limitation. On account of the low price and stable nutrition, plant protein has been considered a promising protein source (Amaya, Davis & Rouse, 2007). Among plant protein-derived candidate species, rice protein has a comparable value of protein and lipid level to fishmeal (Palmegiano et al., 2006), as well as a balanced amino acid profile (Oujifard et al., 2015). Rice protein concentrate has been assessed as a protein source on aquatic species for years (Oujifard et al., 2012; Oujifard et al., 2015; Palmegiano et al., 2006; Palmegiano et al., 2007). However, unlike most marine sources, rice protein lacks small soluble molecular palatable stimulants (NRC, 2011). Hence, it is particularly necessary to improve the palatability of the feed containing rice protein. Squid paste is processed from the organic wastes in squid, and is useful in improving food intake and growth of aquatic species (Amaya, Davis & Rouse, 2007; Hua et al., 2015; Santoso, Ishizuka & Yoshie-Stark, 2013). Thus, to further improve the use of rice protein, squid paste can be appropriately added as an effective enhancer of appetite.

Appetite is controlled by a complex system, in which the gut-brain axis regulates central and peripheral signaling response to nutrient intake. The gastrointestinal tract releases satiety and adiposity signals, including gastric distention and satiation peptides (Powley & Phillips, 2004) such as cholecystokinin (CCK), peptide YY (PYY) and glucagon-like peptide-1 (GLP-1). The signals reach the solitary nucleus (SN) in the caudal brainstem through the vagus nerve. Afterward, the satiety signals combine with the obesity signals (leptin and insulin), as well as multiple hypothalamic and supra-hypothalamic input signals from the NTS afferent fiber projection to the arcuate nucleus (ARC), which forms a complex neural circuits network. Finally, the individual’s reaction to a meal initiates (Valassi, Scacchi & Cavagnini, 2008). As for the neuropeptide system, “first-order” neurons located in ARC secrete the orexigenic neurons, such as co-expressing neuropeptide Y (NPY) and agouti-related peptide (AGRP). These neurons have since been called AGRP neurons, which are inhibited by both insulin and leptin (Cowley et al., 2001; Spanswick et al., 1997), but activated by ghrelin (Cowley et al., 2003). Adjacent to AGRP cells in the ARC is neurons that expressing anorexigenic pro-opiomelanocortin (POMC) and cocaine and amphetamine-regulated transcript (CART). Contrary to AGRP neurons, POMC neurons are stimulated by leptin and are inhibited in the absence of leptin (Fan et al., 1997; Schwartz et al., 1997). Other brain regions controlling food intake are “second-order”, including the paraventricular nucleus (PVN), the lateral hypothalamus (LHA) and perifornical area (PFA). Both gastrointestinal and neuropeptide systems acting in synergism maintain the food intake and energy balance.

Nevertheless, the regulation of food intake on the aquatic animal is quite limited, and the actual research is mainly focused on the gene cloning. Sporadic research indicated that brain-gut peptides in aquatic animals might play a different role in regulatory pathways compared to that in mammals (Cao et al., 2011; Mommsen, 2000; Riley et al., 2008). There is little research on functional analysis of the appetite peptides. As the most critical nutrient to the aquatic animals, protein plays a prominent role in ingestion, growth, and self-healing. Both the amino acid profile (López et al., 2010) and protein level (Coutinho et al., 2012) could affect the food consumption and the expression of appetite peptide, but the opposite results were often observed in the limited reports of aquatic animal (Hevrøy et al., 2008; Sissener et al., 2013). Hence, it is particularly necessary to explore the regulatory mechanism of aquatic animals.

Chinese soft-shelled turtle (*Pelodiscus sinensis*) has been cultivated in Asia for many years because of its high nutritional value and excellent economic benefits. In 2017, *Pelodiscus sinensis* output had been up to 344,529 tons in China (China Fishery Statistical Yearbook, 2018). Nevertheless, the industry is limited by the high feed cost because the cultivation of this species is dependent mainly on fishmeal. Therefore, it is necessary to reduce dietary fishmeal content in this species.

To date, there have been few reports of dietary fishmeal substitution by rice protein and squid paste supplementation in *Pelodiscus sinensis*. Furthermore, less attention has been given to the molecular mechanism of the appetite modulated by the dietary nutrient factor, especially for a reptile. In this context, this study aimed to evaluate feed intake and brain-gut dynamic responses to dietary rice protein concentrate and squid paste in Chinese soft-shelled turtle and shed more light on the molecular mechanism of the appetite when nutrients changes and particularly food is deprived.

## Material and Methods

### Animal ethics

The care and use of animals followed the Animal Research Institute Committee guidelines of Nanjing Agriculture University, China. The Committee has approved this study of the Animal Research Institute of Nanjing Agricultural University, China (permit number: SYXK (Su) 2011-0036).

### Experimental feeds

Three isonitrogenous (47% crude protein) and isoenergetic (18 MJ/kg gross energy) diets were formulated in the present study. The control diet contained 60% fishmeal (CT), while 18% of fishmeal was replaced by rice protein concentrate (RP and RPS) in the test diets. RPS diet was different from the RP diet by the inclusion of 1% squid paste. This concentration of squid paste was determined by the optimal dietary preference of *Pelodiscus sinensis* (Sun et al., 2018a; Sun et al., 2018b). To maintain the consistencies of essential amino acid profiles in the experimental diets, microcapsule lysine was supplemented according to the essential amino acid profiles of the CT diet. The formulation and proximate composition of the experimental diets were presented in Table 1.

**Table 1:** Composition of experimental diet (% dry matter basis).

Ingredients	CT	RP	RPS
White fish meal	60.0	42.0	42.0
Rice protein concentrate^b^	0.0	18.0	18.0
Soybean protein concentrate^c^	7.0	7.0	7.0
Soybean meal^d^	4.0	4.0	4.0
DDGS^e^	4.0	4.0	4.0
Fish oil^a^	1.2	1.2	1.2
*α*-starch^f^	21.1	19.6	18.6
CaH_2_PO_4_	1.4	1.4	1.4
Vitamin and mineral premix^a^	1.3	1.3	1.3
Microcapsule lysine^g^	0.0	1.5	1.5
Squid paste^h^	0.0	0.0	1.0
Proximate composition (%, dry-matter basis)			
Crude protein	46.8	46.7	47.3
Crude lipid	6.5	6.5	6.9
Crude ash	15.2	12.0	11.6
Energy (MJ kg^−1^)	18.1	18.3	18.3

**Notes.**

CT, diets including 60% fishmeal; RP, diets including 42% fishmeal; RPS, diets including 42% fishmeal and 1% squid paste.

a Obtained from Tech-bank Co., Ltd (Ningbo, China).

b Obtained from Hubei Jingyuan Mountain Biotechnology Co., Ltd (Jingmen, China).

c Obtained from Ruilin Biotechnology Co., Ltd (Shanghai, China).

d Obtained from ZhengChang Feed Industry Co., Ltd (Huaian, China).

e Distillers dried grains with soluble, obtained from Qilong Biotechnology Feed Co., Ltd (Shandong, China).

f Obtained fromYinhe Dextrin Co., Ltd (Zhengzhou China).

g Containing 38% lysine was provided by Hainachuan Pharmaceutical Co., Ltd (Foshan, China).

h Obtained from Yancheng Evergreen Conglomerate Co., Ltd (Yancheng, China).

All the ingredients were ground through a 60-mm mesh. The fine powder was carefully weighed, then lipid sources, and 30% of water was added to the mixture that was further blended to ensure homogeneity. A Laboratory pelletizer was used for the pelletizing process. After drying in the laundry drier, the feeds were offered to turtles.

### Turtles and the feeding trial

Juvenile soft-shelled turtles were provided by a commercial farm (Nanjing, China). The feeding trial was conducted from June to July. Turtles were cultured in concrete tanks outside and fed with CT diet for acclimation. After 2-week domestication, turtles of similar size (average 30.65 ± 0.97 g) were randomly distributed into 12 concrete tanks (2.0 m × 2.0 m × 0.8 m), 50 turtles per tank. Three experimental feeds were randomly assigned to turtles with quadruplicate tanks. Management of the feeding trial was conformed to the method of Sun et al. (2018a) and Sun et al. (2018b). Feed pellets were put on a sedentary plate under the water. Turtles were fed approximately 3% of their body weight thrice daily (6:00, 12:00, and 18:00) for eight weeks. This ration was a little bigger than the amount of diet consumed by turtles in 1 h. At each feeding, the uneaten feed was carefully collected by siphoning, dried, and weighed to calculate the total feed intake during the feeding trial. Bodyweight of turtles was measured every two weeks, and the daily feed allowance was adjusted accordingly. Water temperature ranged from 28 to 30 °C, pH fluctuated between 7.2 and 7.4, dissolved oxygen was maintained above 5.0 mg/L, and total ammonia nitrogen and nitrite were kept <0.2 and 0.005 mg/L, respectively, during the feeding trial.

### Sample collection

At the end of the feeding trial, cumulative feed intake per turtle during the feeding trial was calculated as follows: feed intake = cumulative feed consumption/turtle amounts. To determine the effect of short-term food deprivation on gastrointestinal and neuropeptidergic mRNA expression, the turtles were exposed to 48 h food deprivation and then refed *ad libitum* for one hour. We sampled before refeeding (F) and at 3, 6, 12, and 24 h during the postprandial period. Four turtles from each treatment were randomly selected and anesthetized in diluted MS-222 (tricaine methanesulfonate; Sigma) at the concentration of 100 mg/L. The total brain (forebrain, hindbrain, and midbrain) and duodenum were sampled and stored at −80 °C for subsequent analysis. The duodenum begins with pylorus and ends at the suspensory muscle. The turtles were checked before the postprandial harvest. If there were no chyme in its stomach, the turtle would be discarded. We sampled quadruplicate each group per depot. Four operators were allotted to harvest simultaneously to avoid possible variations in mRNA dynamic expression associated with sampling time. Sampling lasted less than 10 mins per group.

### RNA isolation and RT-qPCR analysis

Total brain and duodenum were used for RNA isolation. Total RNA was isolated using RNAiso Plus (Takara Co. Ltd, Japan), and then purified with RNase-Free DNase (Takara Co. Ltd, Japan) to avoid genomic DNA amplification. Purity and concentration of RNA were measured using a NanoDrop (DN-1000, Thermo Scientific, USA). After normalizing the concentration of the RNA samples, cDNA was generated from 500 ng DNase-treated RNA using ExScript™ RT-PCR kit according to the manufacturer’s directions (Takara Co. Ltd, Japan).

The cDNA samples were analyzed by a real-time quantitative detector (BIO-RAD, USA) using the SYBR Green II Fluorescence Kit (Takara Co. Ltd, Japan). The fluorescent qPCR reaction solution consisted of 10µL SYBR® premix Ex Taq™, 0.4µL ROX Reference Dye II, 0.4 µL PCR forward primer (10 µM), 0.4 µL PCR reverse primer (10 µM), 2.0 µL RT reaction (cDNA solution), and 6.8 µL dH_2_O. All RT-qPCR primers were designed using the Primer 5 software and listed in Table 2. The thermal profile was 95 °C for 30s, followed by 40 cycles of 95 °C for 5s and 60 °C for 30s, followed by a melt curve analysis of 15s from 95 to 60 °C, 1min for 60 °C, and then up to 95 °C for 15s. Glyceraldehydes-3-phosphate dehydrogenase (GAPDH) was selected as the housekeeping gene to normalize our samples because of its stable expression in fasting turtle samples in the present study. Values for the threshold (C_T_) from the treated and control tissue templates were compared, and the 2^−ΔΔCT^ method was used as the relative quantification calculation method (Livak & Schmittgen, 2001).

**Table 2:** Nucleotide sequences of the primers used for real-time quantitative PCR.

Gene	GenBank acc. no.	Primer sequences (5′-3′)	Tm (°C)	Amplicon length (bp)
LeptinR	XM_006125027.2	GCCTGCAGGGAATTGGCATA	62	168
		ACAGGCTCCCCACTTGATCG	64	
INSR	XM_014575357.1	ACAACCTCACCATAGCACGG	60	119
		GTCATTCCTCTCCTGACGCC	60	
NPY	XM_006138369.2	TGTCCGTGCTGCCTTTCG	61	146
		GTTGATGTAGTGCCTCAGTGC	59	
POMC	NM_001286918.1	ATGGAACTGGACTACCCCGA	60	91
		TTCCGTCTTTCTTCTCCGCC	60	
CART	XM_014574133.1	ATCGGGAAACTCTGCGACTG	60	121
		AAGGTCACTGGGTCCGTTTG	60	
GLP1R	XM_014575235.1	CAGGGACTGATGGTGGCAAT	60	212
		TAGCTGAATGTGGCTTGGCA	60	
CCK1R	XM_006138180.2	GCAGCAGCACCAAAGTAGAC	59	169
		TCTGCTGATGCGGTGTCAAA	60	
CCK	XM_006131816.2	CCTACACACAGGATAAACG	56	222
		AAACTGACACCAACACAAG	56	
PYY	XM_006118327.1	TCTCTGCCCTCCGACATTAC	59	126
		CGTACCGTGATCTGCCATTG	59	
GAPDH	NM_001286927.1	AGAACATCATTCCAGCATCCA	60	227
		CTTCATCACCTTCTTAATGTCGTC	62	

**Notes.**

LeptinR leptin receptor INSR insulin receptor NPY neuropeptide Y POMC pro-opiomelanocortin CART cocaine and amphetamine-regulated transcript CCK cholecystokinin CCK1R cholecystokinin receptor 1 GLP1R glucagon-like peptide-1 receptor PYY peptide YY GAPDH Glyceraldehydes-3-phosp hate dehydrogenase

One-way analysis of variance (ANOVA) was used to investigate the feed intake. Levene test was used to test the homogeneity of variances. If significant differences were observed (*P* < 0.05), the means were ranked by Tukey’s multiple range test. Two-way ANOVA was adopted to compare the mRNA expression based on diet types, postprandial time, and their interaction. The homogeneity test of variance was performed with the Levene test. The mRNA expression based on one diet type in different postprandial point-in-time and the mRNA expression based on one postprandial point-in-time fed with different diet types were analyzed by one-way ANOVA. If there was a significant difference (*P* < 0.05), the mean was sorted using Tukey’s multiple range test. Analyses were performed using the SPSS program version 16.0 (SPSS Inc., Michigan Avenue, Chicago, IL, USA) for Windows. All data were presented as means ± S.E.M (standard error of the mean).

## Results

### Feed intake for different experimental diets

**Figure 1:**
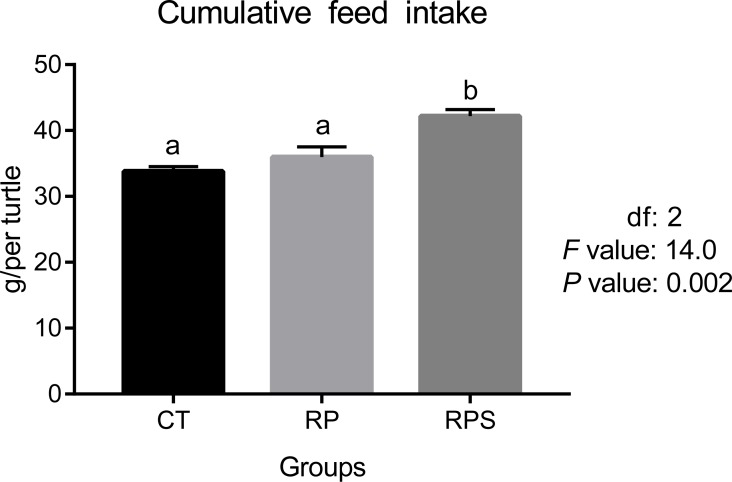
Feed intake in Chinese soft-shelled turtle fed with different experimental diets. CT, diets including 60% fishmeal; RP, diets including 42% fishmeal; RPS, diets including 42% fishmeal and 1% squid paste. Values are means of four replications. Means with different lowercase are significantly different (*P* < 0.05).

According to Fig. 1, feed intake showed no difference when 18% of fish meal was replaced by rice protein concentrate with microcapsule lysine supplemented (*P* = 0.421). However, significantly increased ingestion was observed when 1% of squid paste was included (*P* = 0.02).

### Sequential changes upon fasting and effect of refeeding on INSR and LptinR mRNA levels in the brain and intestinal tract

**Figure 2:**
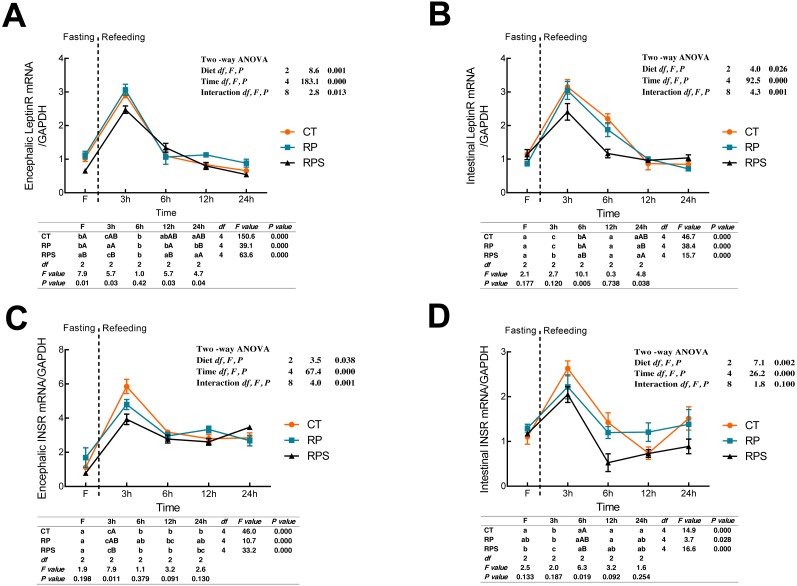
Sequential changes upon fasting and effect of refeeding on INSR and LeptinR mRNA levels in the brain and intestinal tract. (A) The relative mRNA expression of encephalic LeptinR; (B) The relative mRNA expression of intestinal LeptinR; (C) The relative mRNA expression of encephalic INSR; (D) The relative mRNA expression of intestinal INSR. Error bars represent mean ± S.E.M. Different lowercase letters indicate significant differences (*P* < 0.05) at different time points within each treatment, whereas different capital letters indicate significant differences (*P* < 0.05) among these three treatments at each sampling point. CT, diets including 60% fishmeal; RP, diets including 42% fishmeal; RPS, diets including 42% fishmeal and 1% squid paste; LeptinR, leptin receptor; INSR, insulin receptor; GAPDH, Glyceraldehydes-3-phosphate dehydrogenase; ns *P* > 0.05, *P < 0.05, **P < 0.01, ***P < 0.001.

Leptin receptor (LeptinR) mRNA expression in either brain or intestinal tract was affected by the time (*P* < 0.001) and diets (*P* = 0.001, *P* = 0.026) (Figs. 2A and 2B). During fasting in all the depots studied, LeptinR mRNA levels significantly (*P* < 0.05) raised at 3 h fasting and restored to preprandial level at 6–12 h fasting both in the brain and intestinal tract (*P* < 0.001). As for each depot studied, significant differences were observed at F, 3 h, 12 h and 24 h in the brain (*P* = 0.01, *P* = 0.03, *P* = 0.03, *P* = 0.04), as well as 6 h and 24 h in the intestinal tract (*P* = 0.005, *P* = 0.038). Besides, the relative mRNA expression of LeptinR in the brain and intestine was significantly affected by the interaction of diets and time ( *P* = 0.013, *P* = 0.001). As regards to the insulin receptor (INSR) (Figs. 2C and 2D), temporal changes of INSR mRNA expression in the brain and intestine reached a peak at 3 h and then restored to a relatively stable level at 6 h (*P* < 0.001). Upon each depot evaluated, significant changes were only found at 3 h fasting in the brain and 6 h fasting in the intestinal tract (*P* = 0.011, *P* = 0.019). Besides, INSR expression was significantly affected by diets (*P* = 0.038, *P* = 0.002) and time (*P* < 0.001) while the interaction (*P* < 0.01) of diets and time was only found in the brain (*P* = 0.001).

### Sequential changes upon fasting and effect of refeeding on NPY, POMC and CART mRNA levels in the brain

**Figure 3:**
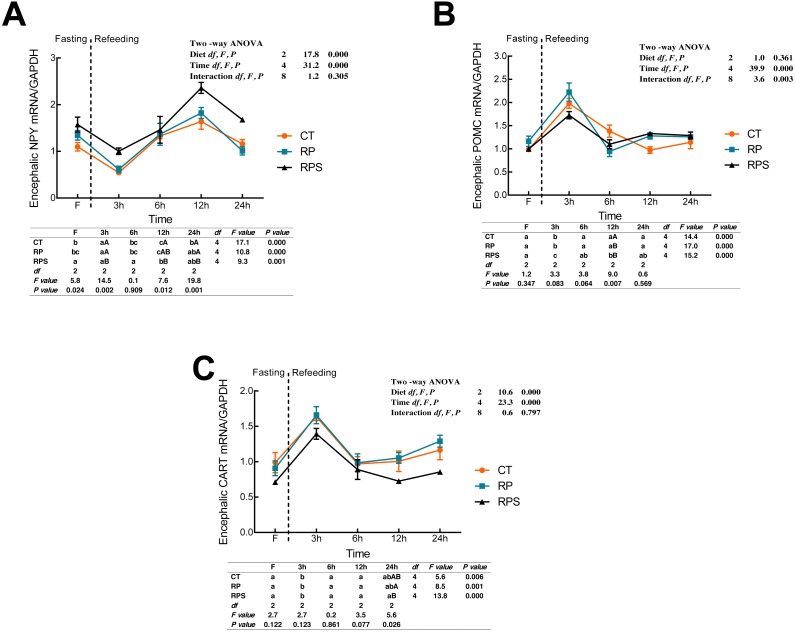
Sequential changes upon fasting and effect of refeeding on NPY, POMC and CART mRNA levels in the brain. (A) The relative mRNA expression of encephalic NPY; (B) The relative mRNA expression of encephalic POMC; (C) The relative mRNA expression of encephalic CART. Error bars represent mean ± S.E.M. Different lowercase letters indicate significant differences (*P* < 0.05) at different time points within each treatment, whereas different capital letters indicate significant differences (*P* < 0.05) among these three treatments at each sampling point. CT, diets including 60% fishmeal; RP, diets including 42% fishmeal; RPS, diets including 42% fishmeal and 1% squid paste; NPY, neuropeptide Y; POMC, pro-opiomelanocortin; CART, cocaine and amphetamine-regulated transcript; GAPDH, Glyceraldehydes-3-phosphate dehydrogenase; ns *P* > 0.05, *P < 0.05, **P < 0.01, ***P < 0.001.

According to Fig. 3A, the relative expression of NPY mRNA in the brain was significantly affected by the diets (*P* < 0.001) and time (*P* < 0.001). With the time-course of fasting, NPY mRNA levels showed a marked reduction at 3 h (*P* < 0.05), and then followed a significant increase as the peaks were observed at 12 h fasting (*P* ≤ 0.001). In terms of each depot, NPY expression in RPS was higher than that in CT and RP group, even though the significant difference was only found at 3 h, 12 h and 24 h fasting ( *P* = 0.002, *P* = 0.012, *P* = 0.001). As shown in Figs. 3B and 3C, dynamic POMC and CART mRNA levels followed a similar tendency with LeptinR and INSR. Contrary to the pattern of NPY, CART expression in RPS was lower than that in CT and RP group, and even a significant difference was only found at 24 h fasting (*P* = 0.026). Furthermore, POMC was significantly affected (*P* < 0.001, *P* < 0.01) by time and the interaction of diets and time (*P* < 0.001, *P* = 0.003), as CART was significantly affected by diets and time (*P* < 0.001).

### Sequential changes upon fasting and effect of refeeding on CCK and CCK1R mRNA levels in the brain and intestinal tract

**Figure 4:**
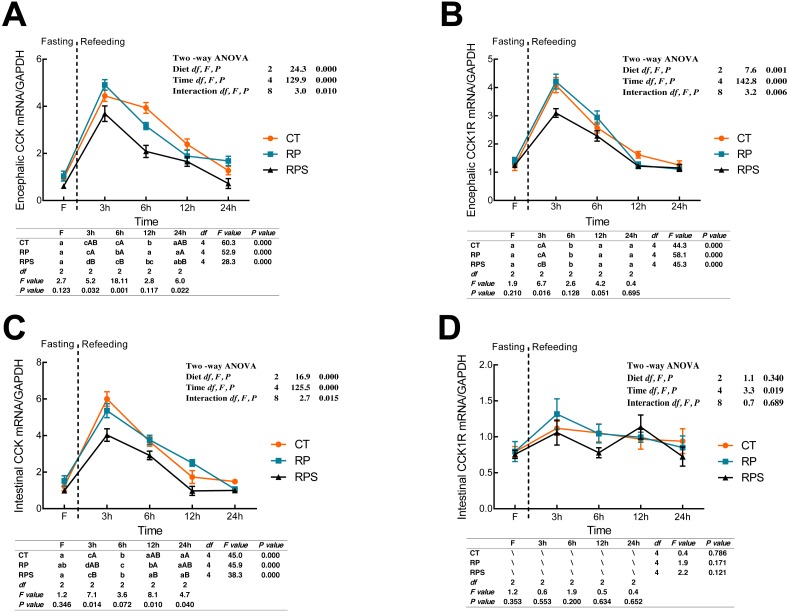
Sequential changes upon fasting and effect of refeeding on CCK and CCK1R mRNA levels in the brain and intestinal tract. (A) The relative mRNA expression of encephalic CCK; (B) The relative mRNA expression of encephalic CCK1R; (C) The relative mRNA expression of intestinal CCK; (D) The relative mRNA expression of intestinal CCK1R. Error bars represent mean ± S.E.M. Different lowercase letters indicate significant differences (*P* < 0.05) at different time points within each treatment, whereas different capital letters indicate significant differences (*P* < 0.05) among these three treatments at each sampling point. CT, diets including 60% fishmeal; RP, diets including 42% fishmeal; RPS, diets including 42% fishmeal and 1% squid paste; CCK, cholecystokinin; CCK1R, cholecystokinin receptor; GAPDH, Glyceraldehydes-3-phosphate dehydrogenase; ns *P* > 0.05, *P < 0.05, **P < 0.01, ***P < 0.001.

CCK and cholecystokinin receptor 1 (CCK1R) mRNA expression were showed in Fig. 4. According to Figs. 4A and 4B, there was a significant increase in CCK and CCK1R mRNA levels of the brain at 3 h postprandial sampling (*P* < 0.05), and continued to drop until 24 h of food deprivation (*P* < 0.001). For each depot studied, the expression of CCK in the brain in RPS reduced significantly at 3 h, 6 h, and 24 h in contrast to that in RP (*P* = 0.032, *P* = 0.001, *P* = 0.022) while significant difference was only found at 3 h fasting in CCK1R expression (*P* = 0.016). In addition, CCK and CCK1R in the brain were significantly affected by diets (*P* < 0.001, *P* = 0.001), time (*P* < 0.001), and the interaction of diets and time (*P* = 0.01, *P* = 0.006). In terms of intestinal CCK mRNA expression (Fig. 4C), intestinal CCK levels increased significantly at 3–6 h fasting and restored to preprandial levels afterward (*P* < 0.001). Additionally, intestinal CCK expression was significantly affected by diets, time, and the interaction of diets and time (*P* < 0.001, *P* < 0.001, *P* = 0.015). As shown in Fig. 4D, CCK1R mRNA levels were only significantly affected by time (*P* = 0.019).

### Sequential changes upon fasting and effect of refeeding on GLP1R and PYY mRNA levels in the brain and the intestinal tract

**Figure 5:**
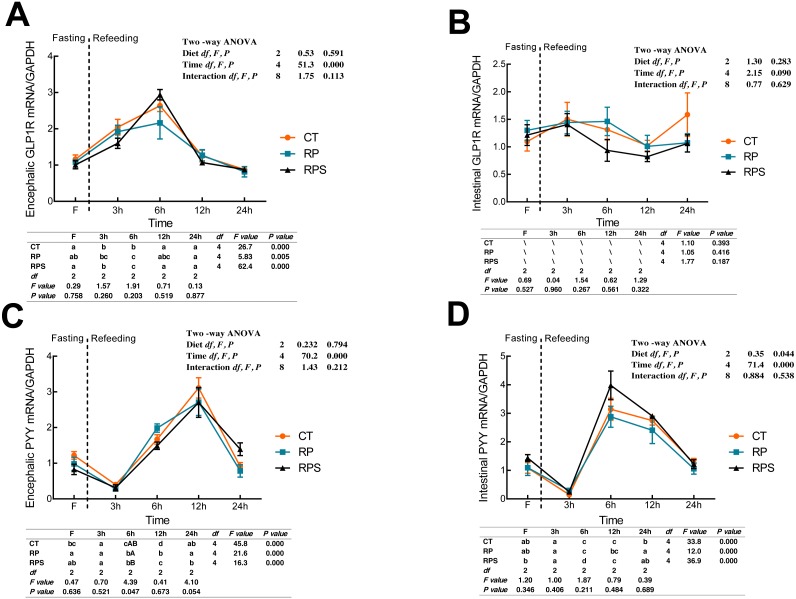
Sequential changes upon fasting and effect of refeeding on GLP1R and PYY mRNA levels in the brain and intestinal tract. (A) The relative mRNA expression of encephalic GLP1R; (B) The relative mRNA expression of intestinal GLP1R; (C) The relative mRNA expression of encephalic PYY; (D) The relative mRNA expression of intestinal PYY. Error bars represent mean ± S.E.M. Different lowercase letters indicate significant differences (*P* < 0.05) at different time points within each treatment, whereas different capital letters indicate significant differences (*P* < 0.05) among these three treatments sat each sampling point. CT, diets including 60% fishmeal; RP, diets including 42% fishmeal; RPS, diets including 42% fishmeal and 1% squid paste; GLP1R, glucagon-like peptide-1 receptor; PYY, peptide YY; GAPDH, Glyceraldehydes-3-phosphate dehydrogenase; ns *P* > 0.05, *P < 0.05, **P < 0.01, ***P < 0.001.

According to Fig. 5A, glucagon-like peptide-1 receptor (GLP1R) in the brain significantly raised its mRNA level at 6 h fasting and then returned to the initial level at 12 h fasting (*P* < 0.001, *P* = 0.005). GLP1R expression in the brain was significantly affected by time (*P* < 0.001), while no statistical difference was found affecting by diets (*P* = 0.591). As with the intestinal CCK1R expression, intestinal GLP1R levels stabilized through the 24 h food deprivation (Fig. 5B). As shown in Figs. 5C and 5D, PYY in both the brain and intestine suffered an unexpected reduction, and afterward, a significant increase in their expression with the time-course fasting (*P* < 0.001). The peaks were observed at 12 h fasting in the brain and 6 h fasting in the gut, respectively. Additionally, PYY mRNA levels in the brain were significantly affected by time (*P* < 0.001) as intestinal PYY mRNA levels were significantly affected by diets and time (*P* = 0.044, *P* < 0.001).

## Discussion

In the present study, dietary rice protein concentrate inclusion showed no effects on feed intake. The results suggested that rice protein concentrate has excellent palatability to *Pelodiscus sinensis*. Similar results were also found in pacific white shrimp (*Penaeus vannamei*), rainbow trout (*Oncorhynchus mykiss*), and blunt snout bream (*Megalobrama amblycephala*) (Cai et al., 2018; Oujifard et al., 2012; Palmegiano et al., 2006). Nevertheless, squid paste showed a significant attractive effect on *Pelodiscus sinensis.* It might be explained by the fact that squid paste is rich in many small molecular substances, such as free amino acid, organic acids, and especially the trimethylamine N-oxide (TMAO). These substances have been turned out to be highly useful to the aquatic animal (Kasumyan & DÖving, 2003; Kohbara et al., 2006; Tian, 2012; Wang et al., 2012). Although the ingestive behavior may be regulated by the synthetic action of these stimulant substances, the physiological regulatory signals response before and after ingestion is scarce in aquatic animals.

Food deprivation means a critical challenge, which must be quickly and harmoniously addressed by different organs to adjust the negative energy balance (Palou et al., 2008). The physiological response to nutritional ingestion during food deprivation provided a comprehensive index of the energy expensed by all activities used to process a diet (Mccue, 2006; Secor, 2009). After a meal, a series of physiological food intake regulation signals occur in the process of digesting, absorbing, and assimilating ingested nutrients (Carter et al., 2001). Thus, the dynamic mRNA expression of the appetitive peptide during short term fasting here could reflect physiological food intake regulation to different nutrition and stimulants.

In the present study, LeptinR and INSR in the brain showed a postprandial increase at 3 h, followed by a significant decrease at 6 h, inferring that LeptinR and INSR in Chinese soft-shelled turtle might induce appetitive peptides directly into the brain to regulate food intake (Campfield et al., 1995; Weigle et al., 1995). It could be supported by the fact that leptin and insulin have a potently inhibiting effect on food intake (Billington, 2001; Porte, Baskin & Schwartz, 2005) and transmit their long-acting signal through LeptinR and INSR in the central nervous system (CNS). Similar results also had been observed in mammals (Baskin et al., 1998). Intestinal LeptinR and INSR showed a similar tendency with that in the CNS. Nevertheless, compared with CT and RP group, turtles fed the diet with the inclusion of squid paste presented a more abrupt decrease with the time course of 12 h fasting. The diet-induced intestinal LeptinR and INSR expression might suggest the cooperation in the intestine on the regulation of food intake between long- and short-acting anorectic signals. It was supported by the emerging evidence that leptin and insulin receptors are expressed on intestinal L cells, which was regarded as the similar synergism between long and short-term signals in the gut. As for different diet types, a significant increase of LeptinR postprandially was also found when feed intake of grass carp (*Ctenopharyngodon idellus*) decreased (Huang et al., 2019). However, the minor effect of leptin was observed in Atlantic salmon (*Salmo salar L.*) fed with different diets at 6 h postprandially, and tilapia (*Oreochromis sp.*) fed with different stimulant at 24 h postprandially (Sissener et al., 2013; Zou et al., 2017). The conflict might attribute to discrepant sampling time and the effects of leptin in different species.

In the present study, POMC and CART expression followed a similar trend with LeptinR and INSR, with a contrary pattern observed in NPY expression. The results might suggest that the endogenous leptin and insulin in Chinese soft-shelled turtle might regulate short-term food intake through the signals to activate specific efferent pathways (NPY or POMC/CART) like mammals. It was supported by the parallel results in red-bellied piranha (*Pygocentrus nattereri*) (Volkoff, 2014), zebrafish (*Danio rerio*) (Nishio et al., 2012), Atlantic salmon (*Salmo salar*) (Valen et al., 2011) and rats (Palou et al., 2009). It could be further justified by the evidence that anorexigenic POMC and CART are stimulated by leptin (Schwartz et al., 1997). At the same time, orexigenic NPY neuron is inhibited, and overlapping signal transduction and transcriptional cascades are activated by insulin (Hill et al., 2010; Spanswick et al., 1997; Spanswick et al., 2000). Additionally, the alteration of NPY and POMC/CART expression in the brain was transient, and their mRNA restored to the preprandial levels at 24 h and 6 h fasting, respectively, inferring that the control power of leptin and insulin is variable to different neuropeptides. NPY in the brain might contribute a more significant long-lasting effect on Chinese soft-shelled turtle. Concerning different diet types, a similar result also found in fish. *Lateolabrax japonicas* fed with palatable diet showed significantly higher mRNA expression of POMC in the hypothalamus than that fed with a control diet at 3 h after feeding (Liang et al., 2019). Decreased expression of CART and increased expression of NPY in the brain was observed when a palatable diet was fed in grass carp and tilapia (Liu et al., 2014; Zou et al., 2017). Therefore, the response of neuropeptides (NPY, CART, and POMC) in *Pelodiscus sinensis* to different diet types might be similar to those in fish.

CCK is produced by I cells in the intestinal mucosa, as well as in the brain and enteric nervous system. CCK interacts with CCK1R and CCK2R expressed in CNS and gastrointestinal tract, in which CCK1R is responsible for ingestion and digestion (Cummings & Overduin, 2007). In the present study, CCK expression in both the brain and intestine improved within 6 h post-meal and reverted to the initial level at 12 h fasting time point, which also followed the parallel pattern of LeptinR and INSR. The results indicated that CCK typified a short-acting satiation signal in *P. sinensis*. Similar results were also observed in mammals (Cummings & Overduin, 2007; Kopin et al., 1999), for example, basal plasma CCK level gradually increase over 10–30 min after meal inhibition and remaining elevated for as long as postprandial 3–5 h in human (Moran & Kinzig, 2004). However, a slight change of intestinal CCK1R expression was observed, while CCK1R mRNA levels showed a similar pattern with CCK in the brain. The inconsistent results might indicate that the regulation of food intake by CCK only acted in CNS for this species. In the present study, turtles fed diets included squid paste presented lower peak mRNA level of CCK and CCK1R than that in other groups. It might be accounted for the improved activities of the digestive enzyme in the RPS group (Sun et al., 2018a; Sun et al., 2018b). Similar results were also found in grass carp (Liu et al., 2014; Huang et al., 2019). However, only a minor decrease of CCK expression in the brain at 15mins after feeding was found when dietary palatability improved to Cobia (*Rachycentron canadum*) (Van Nguyen et al., 2013). The inconsistency might ascribe to the different sampling point-in-time, as warrants further studies.

**Figure 6:**
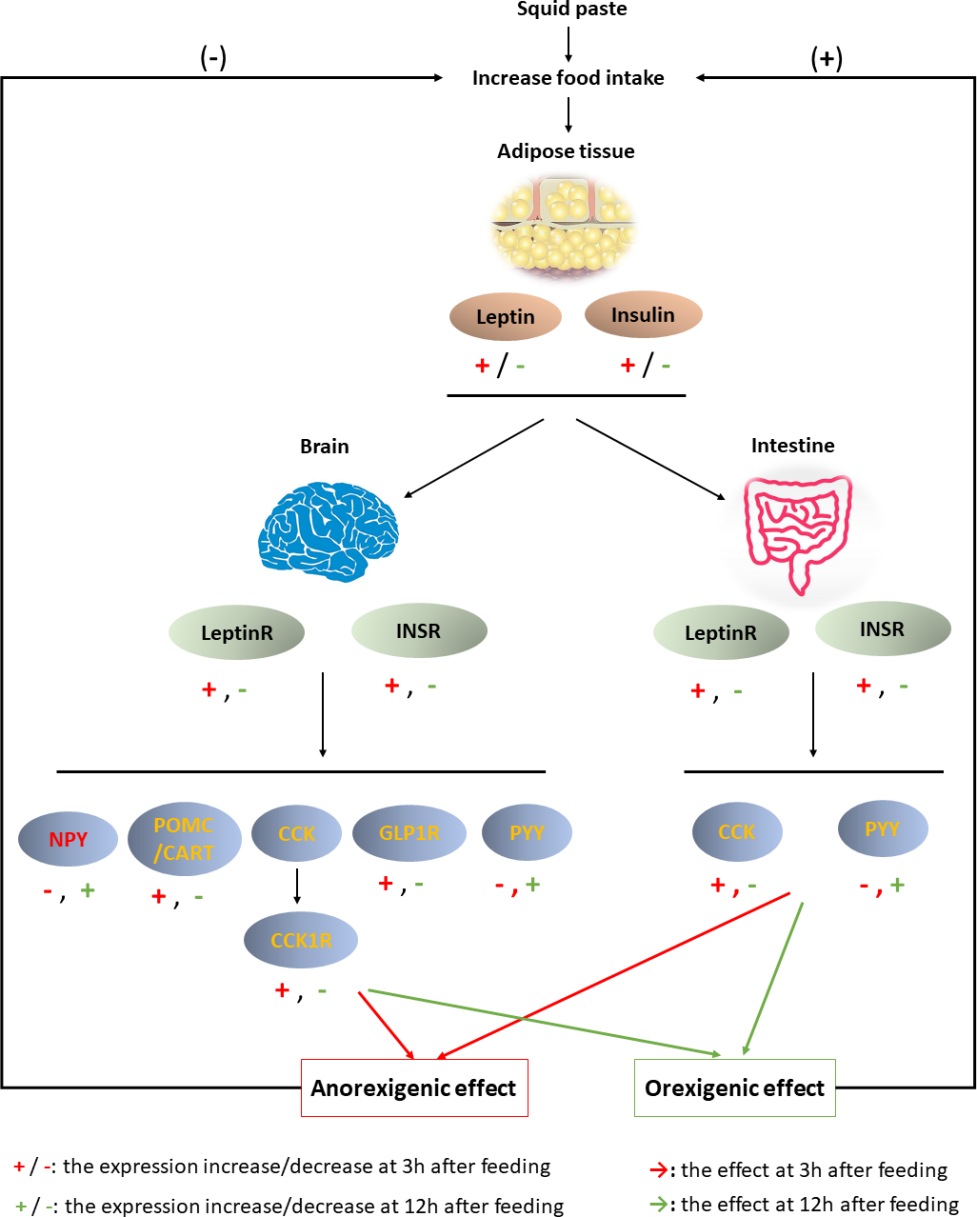
Brain-gut dynamic responses to squid paste during fasting.

GLP1 is secreted primarily by L cells in the colon and distal small intestine. Previous studies have asserted that GLP1 could decrease ingestion with the anorectic effects mediated specifically by GLP1R (Cummings & Overduin, 2007; Donahey et al., 1998; Verdich et al., 2001). In the present study, GLP1R expression in the brain ascended after 6 h fasting and continued to decrease with the time course of fasting, but no significant difference was found in intestinal GLP1R expression. Only GLP1R in the CNS followed a similar tendency with LeptinR and INSR, suggesting that GLP1 induced anorexia possibly directed central pathways. Similar results were also found in mice (*Mus musculus*) (Baggio et al., 2004). It was supported by the fact that LeptinR and INSR expressed on L cells augment GLP1 secretion in either gut or hypothalamus (Anini & Brubaker, 2003). In addition, peripheral GLP1 could be degraded by DPP-IV in the circulation (Orskov, Wettergren & Holst, 1993), which might partly account for the nondiscriminatory GLP1R mRNA level in the intestinal tract. Diet types showed no effects on GLP1R expression in the present study. It might be attributed to that the changed components in the experimental diets were the protein mostly, which showed a negligible effect on stimulating GLP1 secretion compared with lipids and carbohydrates (Brubaker & Anini, 2003; Smeets et al., 2008). As with GLP1, PYY is also produced by distal-intestinal L cells. It delays gastric emptying and promotes the ileal brake (Pironi et al., 1993). Nevertheless, the present study asserted an inconsistent result in that PYY mRNA expression showed an adverse trend with other gastric satiation peptides, such as CCK, POMC, and CART. The results suggested that PYY in Chinese soft-shelled turtle might not be an anorectic peptide. It could be supported by the research that PYY could activate Y1 and Y2 receptors, which evoke the orexigenic effects through the interactions with each other (Batterham et al., 2002). Nevertheless, other reports argued that Y receptors are expressed to medicate NPY-induced feeding, while PYY competitively inhibited the expression of NPY (Kanatani et al., 2000). Thus, PYY decreases ingestion by inhibiting NPY neurons through the Y receptor as NPY has more excellent powerful effects on food intake than PYY (Cummings & Overduin, 2007). Like GLP1, PYY expression in the brain was not affected by diets. It could be partly explained by the fact that the experimental diets were isoenergetic while this peptide is secreted in proportion to caloric load (Degen et al., 2005). However, according to the previous study, postprandial mRNA expression of PYY showed a significant difference in grass carp fed with different diet types (Huang et al., 2019). The conflict might ascribe to different experimental species and nutritional composition of diets, Pending further study.

## Conclusion

In summary, the results obtained here suggested that squid paste is an outstanding stimulant for Chinese soft-shelled turtle. The physiological response to squid paste is shown in Fig. 6. Three hours past feeding, squid paste induced the synthesis of leptin and insulin, which afterward combined with LeptinR and INSR in the brain and intestine, respectively. The anorexigenic peptides, such as POMC, CART, CCK/CCK1R, GLP1R in the brain and CCK in the intestine were activated, while NPY, the orexigenic peptide, was inhibited. Both central and peripheral signals contributed to the anorexigenic effects. Compared with the control group, squid paste led to lower expression of anorexigenic peptides at 3 h past feeding, but higher expression of NPY (orexigenic peptide) at 3 h, 12 h, and 24 h postprandially. These molecular signals in the central and peripheral systems might advance hunger pangs. The changed signals highlight the importance of these peptides and their receptors to short-term food deprivation for this species as well as the effect of squid paste on food intake regulatory mechanism.

##  Supplemental Information

10.7717/peerj.9031/supp-1 Data S1 Raw data of feed intake and mRNA expression

## Additional Information and Declarations

### Data Availability

The following information was supplied regarding data availability: The raw measurements are available in a Supplemental File.
